# Structure-Based Design, Synthesis, and Biological Evaluation of the Cage–Amide Derived Orthopox Virus Replication Inhibitors

**DOI:** 10.3390/v15010029

**Published:** 2022-12-21

**Authors:** Evgenii S. Mozhaitsev, Evgeniy V. Suslov, Daria A. Rastrepaeva, Olga I. Yarovaya, Sophia S. Borisevich, Edward M. Khamitov, Dmitry S. Kolybalov, Sergey G. Arkhipov, Nikolai I. Bormotov, Larisa N. Shishkina, Olga A. Serova, Roman V. Brunilin, Andrey A. Vernigora, Maxim B. Nawrozkij, Alexander P. Agafonov, Rinat A. Maksyutov, Konstantin P. Volcho, Nariman F. Salakhutdinov

**Affiliations:** 1 Department of Medicinal Chemistry, N.N. Vorozhtsov Novosibirsk Institute of Organic Chemistry SB RAS, Lavrentyev Ave. 9, 630090 Novosibirsk, Russia; 2 Laboratory of Chemical Physics, Laboratory of Physical and Chemical Methods of Analysis, Ufa Institute of Chemistry Ufa Federal Research Center, 71 Pr. Oktyabrya, 450078 Ufa, Russia; 3 Synchrotron Radiation Facility SKIF, G.K. Boreskov Institute of Catalysis SB RAS, 630559 Koltsovo, Russia; 4 Scientific Educational Center “Institute of Chemical Technology”, Novosibirsk State University, 630090 Novosibirsk, Russia; 5 State Research Center of Virology and Biotechnology VECTOR, Rospotrebnadzor, 630559 Koltsovo, Russia; 6 Department of Analytical, Physical Chemistry and Polymer Chemistry and Physics, Department of Organic Chemistry, Volgograd State Technical University Lenina, Avenue 28, 400005 Volgograd, Russia; 7 Center of Translational Medicine, Sirius University of Science and Technology, Olympic Avenue 1, Krasnodar Krai, 354340 Sirius, Russia

**Keywords:** adamantane, amides, antipox activity, p37

## Abstract

Despite the fact that the variola virus is considered eradicated, the search for new small molecules with activity against orthopoxviruses remains an important task, especially in the context of recent outbreaks of monkeypox. As a result of this work, a number of amides of benzoic acids containing an adamantane fragment were obtained. Most of the compounds demonstrated activity against vaccinia virus, with a selectivity index SI = 18,214 for the leader compound **18a**. The obtained derivatives also demonstrated activity against murine pox (250 ≤ SI ≤ 6071) and cowpox (125 ≤ SI ≤ 3036). A correlation was obtained between the IC_50_ meanings and the binding energy to the assumed biological target, the p37 viral protein with R^2^ = 0.60.

## 1. Introduction

One of the first infectious diseases defeated by mass vaccination was smallpox, whose causative agent is the variola virus (VARV). Smallpox vaccination has been discontinued since 1980. It is considered that at present more than 50% of the human population does not have immunity against VARV. The World Health Organization considers it necessary to continue to work at finding new low molecules active against VARV [1]. This relates to several reasons, including the possibility of spreading VARV from various types of smallpox burials and the reproduction of VARV or a similar virus for terrorist purposes. [2]. In addition, other orthopoxviruses similar to VARV circulate in nature, such as monkeypox and cowpox viruses, whose mutations can lead to an increase in their pathogenicity for humans. An outbreak of monkeypox in several countries in 2022 brought new public health challenges in addition to the ongoing pandemic of coronavirus disease 2019 (COVID-19). The outbreak has spread to 104 countries on six continents, with the highest incidence in North America and Europe. Monkeypox virus disease has spread rapidly, raising particular concern about human-to-human transmission and community spread in non-endemic regions [3]. Before this, one of the last outbreaks of monkeypox virus diseases in humans was noted in Africa in 2016 [4,5].

**Figure 1:**
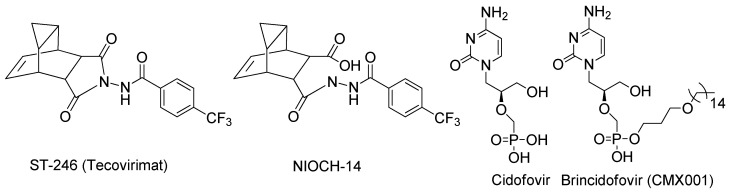
Some compounds currently approved for the treatment of VARV or are in clinical trials.

Currently, ST-246 (Tecovirimat, TPOXX^®^) [6] (Figure 1) is approved in the USA for the treatment of smallpox and monkeypox. ST-246 was developed by SIGA Technologies Inc. (New York, NY, USA). In Russia, NIOCH-14 is officially registered as the antipox drug, whose action mechanism is the same as that of Tekovirimat. It should be noted that NIOH-14 is a prodrug of ST-246 [7].

Another smallpox drug, CMX001 (Brincidofovir), was approved by the US FDA in June 2021 for the treatment of smallpox in humans [8,9]. CMX001 is a lipophilic nucleotide analogue of Cidofovir (Cidofovir, CDV, Vistide^®^), which is an antiviral drug used for the treatment of cytomegalovirus retinitis, active in lethal models of orthopoxvirus infection in mice and monkeys [10] (Figure 1). Low oral bioavailability and significant nephrotoxicity were demonstrated for the Cidofovir. Moreover, it was shown to be ineffective when used after the onset of smallpox lesions in VARV-infected monkeys. In the case of CMX001, it was found that it does not provide a sufficient level of protection against a lethal infection of mice with the ectromelia virus in experiments with small rodents [11].

In 2010, the World Health Organization (WHO) published the report “Scientific Review of Research on Variola Virus 1999–2010” [10] and comments of the WHO Advisory Group of Independent Experts on Smallpox Programme Review (AGIES) concerning this issue [12]. AGIES experts noted that VARV was experimentally shown to develop resistance to CMX001 and Tecovirimat and further investigations are necessary to find new agents with different mechanisms of action.

**Figure 2:**
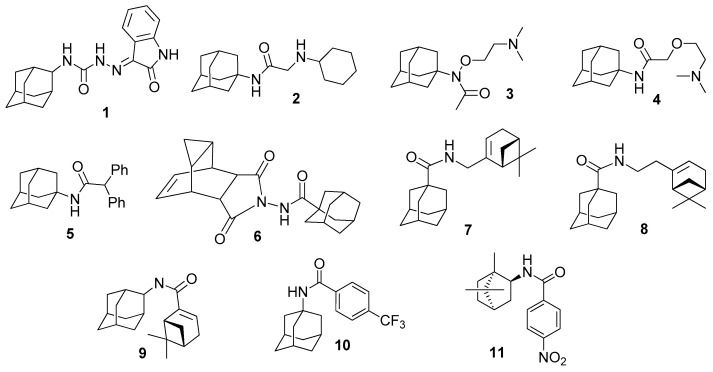
A number of adamantane or bicyclic monoterpene derivatives (**1**–**11**), active against orthopoxviruses.

Compounds containing an adamantane fragment demonstrated a wide range of biological activity, including antiviral activity [13]. In particular, semicarbazone **1** containing an isatin fragment, was found to be a vaccinia virus inhibitor (Figure 2), leading to a 42% reduction in vaccinia virus reproduction after a three-hour exposure in cell culture at 2 μg/mL concentration [14]. Some secondary and tertiary amines containing an adamantane fragment, such as compounds **2, 3**, and tromantadine **4**, demonstrated pronounced vaccinia virus inhibitory effects [15]. It was shown in [16] that diphenyl derivative amide **5** inhibits vaccinia virus reproduction in cell culture in the range from 72% to 100% at a concentration of 2 µg/mL. However, the activity of these compounds against other types of orthopoxviruses was not investigated. Interestingly, there are data concerning the activity of Tecovirimat derivative 6 on orthopoxviruses obtained by replacing the aromatic fragment with adamantyl [17]. For **6**, it was shown that concentrations below 0.05 μM are effective against vaccinia and cowpox viruses.

The activity of 1- and 2-aminoadamantane amides containing monoterpene acid fragments against vaccinia, cowpox, and mousepox viruses was shown in [18]. The highest activity simultaneous with low toxicity was demonstrated for amides **7**–**9** (Figure 2). The highest selectivity indices (SI), i.e., the ratio of TC_50_ (test compound concentration at which 50% of cells in the non-infected monolayer are destroyed) to IC_50_ (concentration of the compound at which 50% of cells in the infected monolayer are not destroyed and remain viable) were shown for **7**, being from 406 (for cowpox virus) to 1123 (for vaccinia virus). At the same time, the selectivity index for the vaccinia virus in the reference drug cidofovir was 12. It should be noted that the exhibited activity critically depended on the type of terpene substituent [18].

On the other hand, introduction of aromatic fragments instead of monoterpene fragments into the compounds seems to be a promising approach, in particular due to the relatively greater availability of the starting compounds. It is important that the structure of Tecovirimat, which demonstrates a high activity against poxviruses, contains a fragment of 4-trifluoro-substituted benzoic acid. In [19], the synthesis and investigation of the activity against orthopoxviruses for a wide range of aromatic amides containing adamantane fragments were described with the selectivity index of 2325 in the case of the **10**.

It is also necessary to mention the article [20], which describes a synthesis of a number of derivatives, combining fragments of benzoic acids and camphor or fenchone scaffolds (adamantane bioisosteres). In particular, for amide of an *endo*-bornylamine derivative **11**, a selectivity index of 31,500 was shown.

Molecular modeling of interactions between the adamantane amide derivatives and the proposed molecular target—the p37 protein encoded by the F13L gene [21]—was performed in [19]. The p37 protein plays an important role in the viral envelope formation and the virus release from the host cell [22,23,24]. For the protein, a wide spectrum of lipase activity was shown as well [25]. The p37 was previously shown to be the molecular target of Tecovirimat [26], however, its mechanism of action and the binding site with p37 were not elucidated. Nevertheless, in [19] the binding site was chosen based on the molecular modeling results and the assumption that p37 exhibits phospholipase activity. In particular, based on the presence of the HKD domain (H—histidine; K—lysine; D—asparagine) in p37, this protein was compared with phospholipases and the binding site was used to describe the antiviral activity mechanism of a number of compounds containing an adamantane fragment. On the other hand, the adamantane fragment is similar geometrically and by lipophilicity to the cage fragment of Tecovirimat, which can determine the adamantane derivatives activity against orthopoxviruses.

Thus, the data indicates the importance of further investigations aimed at the search for new, safe, and effective agents active against orthopoxviruses. The goal of this work was to synthesize both previously described and new amides of aromatic carboxylic acids containing adamantane fragments and to investigate their biological activity against a wide range of orthopoxviruses. Special attention was paid to the crystal structures of the synthesized substances and discussion of the proposed action mechanism of the compounds.

## 2. Materials and Methods

### 2.1. Chemistry

#### 2.1.1. General Information

All chemicals were purchased from commercial vendors and used without further purification, unless indicated otherwise. ^1^H- and ^13^C-NMR spectra were registered on a Bruker Avance—III 400 (400.13 MHz (^1^H) and 100.71 MHz (^13^C) in CDCl_3_). Chemical shifts obtained are given in ppm, relative to residual chloroform (δ_H_ 7.24, δ_C_ 76.90 ppm), and J are given in Hz. Numeration of atoms in the compounds (see Supplementary Materials, Figure S1) is given for assigning the signals in the NMR spectra and does not coincide with the nomenclature of compounds. The elemental composition of compounds was determined from high-resolution mass spectra (HR-MS) recorded on a DFS Thermo Scientific spectrometer in full scan mode (0–500 m/z, 70 eV electron impact ionization, direct sample injection). The conversion of reagents and the purity of the target compounds were determined using gas chromatography methods: 7820A gas chromatograph (Agilent Tech., Santa Clara, CA, USA), flame-ionization detector, HP-5 capillary column (0.25 mm × 3 m × 0.25 μm), helium carrier gas (flow rate 2 mL/min, flow division 99:1), temperature range from 120 °C to 280 °C, heating of 20 °C/min. The purity of the target compounds for biological testing was confirmed to be more than 95%.

Suitable for XRD crystals of **12a**, **12b**, **15a**, **18a**, **16b**, **17b** were obtained by slow evaporation of Et_2_O solution; **14a**, **16a**, **12b**, **13b**, **18b** were obtained from hot EtOH solution and **17a** was obtained from hot toluene solution.

XRD of **12a**–**18a**, **12b**, **13b**, **15**–**18b** was performed on Oxford Gemini R Ultra (Rigaku Oxford Diffraction) with Mo*Kα* (λ = 0.71073 Å), graphite monochromator, and CCD detector. The reflex intensities were measured by ω-scanning of frames (1°). Absorption correction was applied using CrysAlis^Pro^ v.1.171.41.93a [27] by the multi-scan method. The structures were solved and refined using SHELXS [28] and SHELXL [29] with the help of Olex2 version 1.5 [30] software. All non-hydrogen atoms were refined in anisotropic approximation. Hydrogen atom positions were refined freely, anisotropic displacement parameter of hydrogen atoms was U_iso_(H) = 1.2·U_eq_(atom). Nitro group of **17b** structure was disordered, for O2B of a nitro group ISOR was performed, O3B-N2, O3A-N2, N2-O2B, N2-O2A distances were restrained by SADI, as well as for N2-C16-O3B-O2B fragment FLAT was performed with standard deviation. XRD data have been deposited with the Cambridge Structural Database (CCDC) (see Supporting Information, Tables S1–S3) and are available from the authors or at the address: http://www.ccdc.cam.ac.uk/conts/retrieving.html (accessed on 1 December 2022).

#### 2.1.2. General Method of Synthesis of Amides **12a**,**b**–**18a**,**b**

To solutions of 1 eq corresponding benzoic acids chlorides and 2 eq of triethylamine in dry toluene, 1 eq of 1- or 2-aminoadamantanes hydrochlorides was added at 0 °C. The resulted mixtures were stirred for 1 h at 0 °C and 12 h at room temperature. After solvent evaporation, the mixtures were suspended in EtOAc and treated with 5% HCl, 5% NaOH, and saturated NaCl water solutions consequentially with following drying over sodium sulfate. The resulted amides were isolated by crystallization from Et_2_O. NMR data of **13a**,**b**–**16a**,**b**, **19a** agree with the literary ones: **12a** [31], **12b [32]**, **13a [33]**, **13b [34]**, **14a [35]**, **14b [36]**, **15a [37]**, **15b [36]**, **18a [38]**.

#### 2.1.3. N-(adamantan-1-yl)-2-nitrobenzamide **16a**

Yield 70%. ^1^H-NMR (CDCl_3_): 1.69 (s, 6H, 2H-4, 2H-6, 2H-10); 2.10 (s, 9H, 2H-2, 2H-8, 2H-9, H-3, H-5, H-7); 5.44 (br. s, 1H, H-11); 7.42–7.52 (m, 1H, H-17); 7.50–7.55 (m, 1H, H-18); 7.57–7.66 (m, 1H, H-16); 7.95–8.05 (m, 1H, H-15). ^13^C-NMR (CDCl_3_): 52.77 (C-1), 41.33 (C-2, C-9, C-8) 29.24 (C-3, C-5, C-7), 36.07 (C-4, C-10, C-6), 163.92 (C-12), 137.43 (C-13), 147.84 (C-14), 121.30 (C-15), 129.56 (C-16), 132.96 (C-17), 125.47 (C-18). HR MS: 300.1464 (*M^+^*, C_17_H_20_O_3_N_2_^+^; calculated 300.1468).

#### 2.1.4. N-(adamantan-2-yl)-2-nitrobenzamide **16b**

Yield 57%. ^1^H-NMR (CDCl_3_): 1.61–1.71 (m, 2H, H-4, H-9); 1.71–1.79 (m, 4H, H′-4, 2H-6, H′-9); 1.76–1.93 (m, 6H, H-5, H-7, 2H-8, 2H-10); 2.04–2.13 (m, 2H, H-1, H-3); 4.17–4.32 (m, 1H, H-2); 6.02–6.19 (br. d, 1H, *J*(11, 2) = 6.8 Hz, H-11); 7.47–7.52 (m, 1H, H-18); 7.51–7.58 (m, 1H, H-17); 7.61–7.68 (m, 1H, *J*(16, 17) = 8.04 Hz, H-16); 7.99–8.07 (m, 1H, H-15). ^13^C-NMR (CDCl_3_): 31.39 (C-1, C-3), 53.96 (C-2), 31.69 (C-4, C-9), 26.87 and 26.93 (C-5, C-7), 37.24 (C-6), 36.88 (C-8, C-10), 165.54 (C-12), 133.25 (C-13), 146.18 (C-14), 124.34 (C-15), 130.10 (C-16), 133.55 (C-17), 128.63 (C-18). HR MS: 300.1464 (*M^+^*, C_17_H_20_O_3_N_2_^+^; calculated 300.1468).

#### 2.1.5. N-(adamantan-1-yl)-3-nitrobenzamide **17a**

Yield 37%. ^1^H-NMR (CDCl_3_): 1.68–1.73 (m, 6H, 2H-4, 2H-6, 2H-10); 2.07–2.17 (m, 9H, 2H-2, 2H-8, 2H-9, H-3, H-5, H-7); 5.94–5.83 (br. s, 1H, H-11); 7.60 (t, 1H, H-17); 8.05–8.11 (dm, 1H, *J*(18, 17) = 7.84 Hz, H-18); 8.26–8.33 (dm, 1H, *J*(16, 17) = 7.84 Hz, H-16); 8.46–8.51 (m, 1H, H-14). ^13^C-NMR (CDCl_3_): 52.77 (C-1), 41.33 (C-2, C-9, C-8) 29.24 (C-3, C-5, C-7), 36.07 (C-4, C-10, C-6), 163.92 (C-12), 137.43 (C-13), 121.31 (C-14), 147.84 (C-15), 125.47 (C-16), 129.56 (C-17), 132.96 (C-18). HR MS: 300.1470 (*M^+^*, C_17_H_20_O_3_N_2_^+^; calculated 300.1468).

#### 2.1.6. N-(adamantan-2-yl)-3-nitrobenzamide **17b**

Yield 34%. ^1^H-NMR (CDCl_3_): 1.68–1.74 (m, 2H, H-4, H-9); 1.75–1.86 (m, 4H, H′-4, 2H-6, H′-9); 1.87–1.96 (m, 6H, H-5, H-7, 2H-8, 2H-10); 2.00–2.10 (m, 2H, H-1, H-3); 4.2–4.29 (m, 1H, H-2); 6.38–6.55 (br. d 1H, *J* (11, 8) = 6.15 Hz, H-11); 7.63 (t, 1H, H-17); 8.08–8.16 (dm, 1H, *J* (18, 17) = 7.73 Hz, H-18); 8.29–8.37 (dm, 1H, *J* (16, 17) = 8.04 Hz, H-16); 8.50–8.59 (m, 1H, H-14). ^13^C-NMR (CDCl_3_): 31.68 (C-1, C-3), 54.08 (C-2), 31.9 (C-4, C-9), 26.87 and 26.98 (C-5, C-7), 37.24 (C-6), 36.9 (C-8, C-10), 164.17 (C-12), 136.35 (C-13), 121.54 (C-14), 148.01 (C-15), 125.76 (C-16), 129.72 (C-17), 133 (C-18). HR MS: 300.1473 (*M^+^*, C_17_H_20_O_3_N_2_^+^; calculated 300.1468).

#### 2.1.7. N-(adamantan-2-yl)-4-nitrobenzamide **18b**

Yield 79%. ^1^H-NMR (CDCl_3_): 1.66–1.74 (m, 2H, H-4, H-9); 1.75–1.85 (m, 4H, H′-4, 2H-6, H′-9); 1.86–1.95 (m, 6H, H-5, H-7, 2H-8, 2H-10); 2.01–2.08 (m, 2H, H-1, H-3); 4.2–4.27 (m, 1H, H-2); 6.38–6.52 (br. d, 1H, *J* (11, 8) = 6.41 Hz, H-11); 7.86–7.95 (dm, 2H, *J* (14,15) = *J* (18, 17) = 8.75 Hz, H-14, H-18); 8.2–8.31 (dm, 2H, *J* (15,14) = *J* (17, 18) = 8.7 Hz, H-15, H-17). ^13^C-NMR (CDCl_3_): 31.71 (C-1, C-3), 54.07 (C-2), 31.94 (C-4, C-9), 26.87 and 27.01 (C-5, C-7), 37.24 (C-6), 36.9 (C-8, C-10), 164.57 (C-12), 140.77 (C-13), 127.94 (C-14, C-18), 123.73 (C-15, C-17), 149.32 (C-16). HR MS: 300.1471 (*M^+^*, C_17_H_20_O_3_N_2_^+^; calculated 300.1468).

### 2.2. Biology

Cytotoxicity and antiviral activity of synthesized amides against vaccinia, cowpox, and mousepox (ectromelia) viruses were evaluated using an adapted colorimetric method in Vero cell culture [39]. The vaccinia virus (strain Copenhagen), cowpox virus (strain Grishak), mousepox virus—ectromelia (strain K-1), obtained from the State collection of pathogens of viral infections and rickettsioses of the SRC VB Vector (Koltsovo, Novosibirsk region, Russia) were used in this work. Viruses were produced in Vero cell culture in DMEM medium (BioloT, Russia). The concentration of viruses in the culture fluid was determined by the method of plaques by titration of the samples in Vero cell culture, calculated and expressed in decimal logarithms of plaque-forming units per ml (log10 PFU per ml). The concentration of the virus in the samples used in this work was from 5.6 to 6.1 log10 PFU per mL. The used series of viruses with the indicated titer was stored at −70 °C. The antiviral efficacy of compounds was evaluated according to the adapted and modified method [39]. The commercially available drug Cidofovir (Cidofovir, Vistide) manufactured by GileadSciencesInc. was used as a reference (Foster City, CA, USA). To evaluate the antiviral activity, firstly, a 50 μL dilution of samples was added to the wells of 96-well plates with a monolayer of cells containing 100 μL of DMEM medium with 2% fetal serum, and then 50 μL of a 1000 PFU per well virus dose was added. The cytotoxicity of compounds was determined based on cell destruction under the derivative influence in the wells, into which no virus was introduced. Cell monolayers in the plate wells were used as controls, into which the virus without compounds was added (virus control) and cell monolayers in the wells, into which neither the virus nor the compound was added (cell culture control). After the incubation of cell monolayers infected with orthopoxvirus and treated with the tested compounds for 4 days, a vital dye “neutral red” was added to the culture medium for 1.5 h. Next, the monolayer was washed twice with saline solution, the lysis buffer was added and after 30 min the optical density (OD), which is an indicator of the number of cells in the monolayer not destroyed in the presence of the virus, was measured on an Emax plate reader (Molecular Devices, San Jose, CA, USA) at 490 nm. The OD values were used to calculate a 50% cytotoxic concentration (CC_50_ μM) and a 50% virus inhibiting concentration (IC_50_ μM) using the SoftMax Pro-4.0 computer program. Based on these indicators, the selectivity index (SI) was calculated: SI = TC_50_/IC_50_.

### 2.3. Molecular Modeling

All theoretical calculations were carried out using software Schrodinger Small Molecule Drug Discovery Suite 2022-1 [40]. The geometric parameters of the ligands were optimized in the OPLS4 [41] force field considering all possible conformations. To perform the AIM analysis, amides **16a**,**b** were additionally optimized at the DFT level using M062 functional [42] with GD3 dispersion correction [43] and 6-311 + G(d,p) basis set [44]. The AIM analysis and estimation of hydrogen bonds strength was carried out using software Multiwfn version 2.1.2 [45].

For molecular modeling, we used geometric parameters of the p37 protein obtained as a result of folding [20]. We considered the cavity in the protein as a proposed binding site, as described in [19,20]. In particular, Phe52, Leu118, Cys120, Ser135, Asn312, Lys314, Asn329, and Asp331 amino acids were considered to form the binding site. For molecular docking we used the reference ligand–protein model (ST-246 [26,46]—p37), obtained as a result of molecular modeling described in [20]. Molecular docking was performed using the forced ligand positioning protocol (IFD) [47,48,49] with the following conditions: flexible protein and ligand; grid matrix size of 20 Å; amino acids (within a radius of 5 Å from the ligand) restrained and optimized, taking into account the influence of the ligand; the maximum number of positions was limited to 20; docking solutions were ranked by evaluating the following calculated parameters: docking score (based on Glide score minus penalties); parameter of model energy value (Emodel), including Glide score value, energy unrelated interactions, and the parameters of energy spent on formation of the laying of the compound in the binding site and binding energy of ligand and protein (IFD score). The absence or minimum number of unfavorable clash interactions was taken into account during the analysis of protein–ligand interactions. Binding energies (ΔG_MM-GBSA_) for ligand–protein complexes were estimated using variable-dielectric generalized Born model for best docking positions, with water taken as a solvent. The influence of amino acid residues located at a distance of 5 Å on the ligand was considered.

## 3. Results and Discussion

### 3.1. Organic Synthesis

**Scheme 1:**
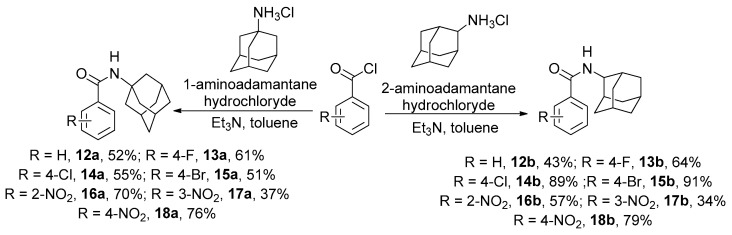
Synthesis of amides **12a**,**b**–**18a**,**b**.

Firstly, the synthesis of a number of aromatic amides containing adamantane fragments was performed. Substances **12a**,**b**–**18a**,**b** were obtained by interaction of substituted aromatic carboxylic acid chlorides with 1- and 2-aminoadamantane hydrochlorides in toluene in the presence of two excesses of triethylamine (Scheme 1). After reaction mixture treatment and crystallization of the products from diethyl ether no additional purification was needed.

It should be mentioned that the synthesis of some of the obtained amides was previously described in [31,32,33,34,35,36,37,38].

**Scheme 2:**
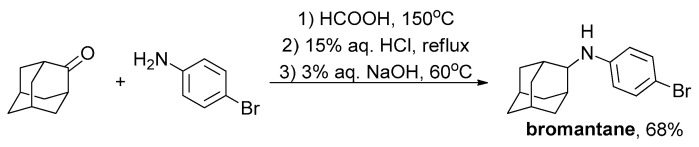
Bromantane synthesis.

To reveal the influence of the linker type between the adamantane fragment and the aromatic one, bromantane was synthesized by reductive amination of adamantan-2-one with 4-bromoaniline under the conditions of the Leuckart–Wallach reaction according to the procedure [50] with a yield of 68% (Scheme 2).

### 3.2. X-ray Analysis of Single Crystals

**Figure 3:**
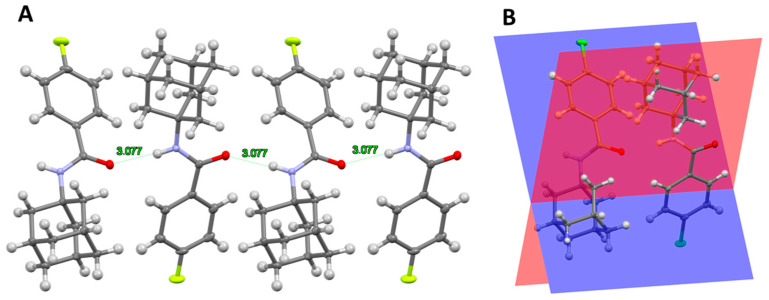
(**A**) A partial packing plot of **13a** showing zigzag chains along the **c**-axis direction resulting from weak N-H⋯O contacts; (**B**) a partial packing plot of **14a** showing the angle of deviation between the planes that pass through the aromatic rings.

The structures of all compounds except **12a** [51] were determined for the first time. Compounds **12a**–**15a** were crystallized in orthorhombic space group *P*bca and they were isostructural. Their molecular structure consists of an adamantan-1-yl group, attached to N benzamides with different substituents in the 4th position: hydrogen, fluorine, chlorine, and bromine. Cell packing shows zigzag chains along the ***c***-axis direction resulting from weak N-H⋯O hydrogen bonds (Figure 3A). The molecules are staggered and the angle of deviation between the planes that pass through the aromatic rings is 47.6°, 48.5°, 56.4°, and 58.7° for **12a**, **13a**, **14a**, and **15a**, respectively (Figure 3B).

**Figure 4:**
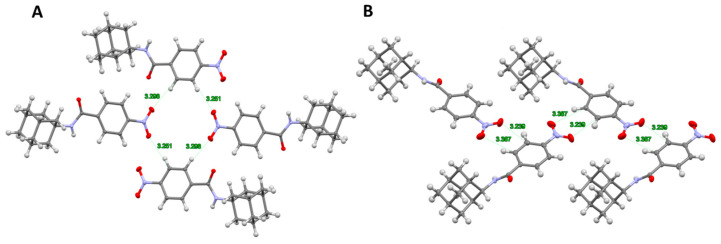
A partial packing plot of **18a** (**A**) and **18b** (**B**) showing a short contact between 4-NO_2_-group and H-atoms neighboring molecules.

Compounds **13a**, **16a**, **16b**, **17b**, **18a**, and **18b** were crystallized in the monoclinic space group *P*2_1_/c, **17a** was crystallized in the non-centrosymmetric space group Pc, and **12b** was crystallized in the triclinic space group *P*-1. The asymmetric unit cell of **12b** and **17a** contains 4 and 2 molecules, and the others have one independent molecule. All structures except **17a** have N-H⋯O contacts which can be classified as weak hydrogen bonds. For these compounds, the N⋯O distance is from 2.971 to 3.151 Å, while in **17a** these contacts have lengths of 3.254 Å and 3.262 Å. 4-F and 2-NO_2_ groups of **13b**, **16a**, and **16b** compounds are not involved in the formation of hydrogen bonds or short contact. The 3-NO_2_ group of **17a** and **17b** has weak O-π interactions with the aromatic ring neighboring molecule. Compounds **13a**, **14a**, and **15a** have halogen⋯π interaction, **17a** and **17b** have π-π interaction. Compounds **18a** and **18b** form a short contact between 4-NO_2_-group and H-atoms neighboring molecules of two different types: in **18a** a non-planar cycle of four molecules and in **18b** a planar network of multiple molecules are formed (Figure 4a,b). The crystal structures of all compounds have a lot of different types of intermolecular interactions and most of these structures (except **12a**–**15a**) have different spatial organization.

The X-ray analysis of single crystals is a widely used method to determine crystal structures of subsequent compounds with atomic resolution. These data can be used in future investigations of drug design by modifying the structure to increase the bioavailability of substances and polymorph screening. The performed experiments allow us to obtain atomic coordinates of the studied molecules, thus, to verify structure optimization and docking results.

### 3.3. Biology

The activity of the obtained compounds against orthopoxviruses was investigated using vaccinia viruses (Copenhagen strain); the values of inhibitory activity (IC_50_) and cytotoxicity (TC_50_) were evaluated using an adapted colorimetric method in Vero cell culture [39] (Table 1). Cidofovir (Heritage Consumer Products, LLC, East Brunswick, NJ, USA) was used as a positive control.

It should be noted that **15b** was previously investigated for activity against orthopoxviruses, in particular, against the vaccinia virus, as well as the cowpox and ectromelia viruses [19] with IC_50_ of 3.06 µM and TC_50_ > 299.2 µM. In our experiments, the activity of **15b** was confirmed, and even better IC_50_ and TC_50_ values were obtained.

Most of the studied compounds showed significant activity against the vaccinia virus with IC_50_ of up to 0.03 µM for **18a**. Isomeric derivatives of adamantane substituted at the 1- and 2-positions showed similar activity, a significant difference was obtained only in the case of **13a**,**b**. However, there was a slight trend towards an increase in the activity of 1-aminoadamantane derivatives. In most cases, except for derivatives of meta-nitrobenzoic acid **17a**,**b**, 1-aminoadamantane amides were shown to be significantly less cytotoxic than isomeric derivatives of 2-aminoadamantane. Thus, these trends led to a significantly higher selectivity index for 1-substituted adamantane derivatives **12a**–**15a**, **18a**.

Regarding the substitution position of the aromatic fragment, it can be noted that the compounds containing the aromatic fragment with *para*-substituents showed the highest activity, in particular, when comparing unsubstituted derivatives **12a**,**b**, as well as nitro derivatives **16a**,**b**–**18a**,**b**. At the same time, chlorine and bromine derivatives demonstrated similar activity (IC_50_ equal to 0.13–0.18 µM), while nitro-derivatives **18a**,**b** were found to be almost five times more active (IC_50_ equal to 0.03 and 0.04 µM) and significantly less cytotoxic than *meta*-nitro substituted amides **17a**,**b**, which led to the highest selectivity indices of 18,214 and 12,300, respectively.

**Figure 5:**
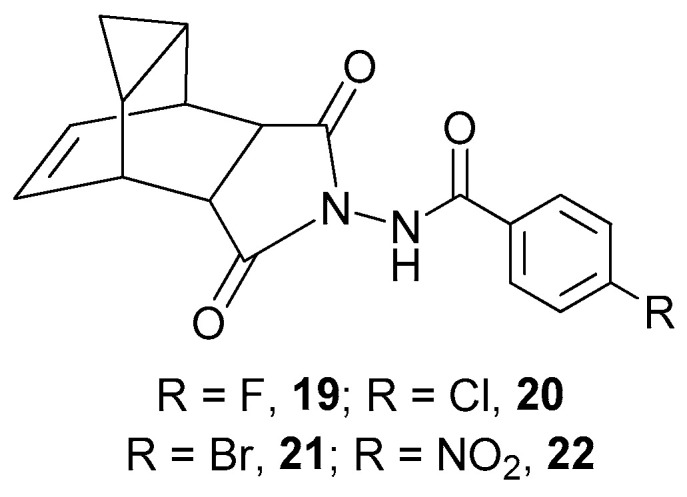
Tecovirimat derivatives **19**–**22** with aromatic substituent, contained in **13a**,**b**–**15a**,**b, 18a**,**b**.

It should be noted that the obtained compounds are similar to Tecovirimat in their structure; in particular, they contain a bulky lipophilic scaffold and an aromatic fragment. Interestingly, the synthesis and investigation of activity against poxviruses of Tecovirimat analogues containing 4-fluorine **19**, 4-chloro- **20**, 4-bromo- **21**, and 4-nitro-phenyl **22** fragments (Figure 5) are described in [17,52] with **19–22** being structurally similar to derivatives **13a**,**b**–**15a**,**b**, **18a**,**b**.

The Tecovirimat analogs **19–22** demonstrated activity against vaccinia virus with IC_50_ < 0.05 μM for **19**, and IC_50_ of 0.02 μM for **20**–**22**. The obtained adamantane derivatives showed generally less activity except the compound **18a** with IC_50_ of 0.03 µM, which is similar to the IC_50_ of the Tecovirimat analog **22**. At the same time, **18a** is much easier to synthesize than **22**.

It is also worth noting that amides of 4-nitrobenzoic acid **18a**,**b** demonstrated the highest activity, which also agreed with the results obtained in [20], where amide **11** with SI = 31,500 showed the highest activity (Figure 2).

Comparing bromantane with structurally similar 4-bromine substituted amide **15b** one may conclude that bromantane was found to be significantly less active (IC_50_ of 0.22 µM for **15b** against 38.8 µM for bromantane) with higher cytotoxicity (TC_50_ of 129.67 µM for **15b** against 86.6 µM for bromantane). It seemed possible that acyl linker led to an increase in activity, but at the same time the linked fragments played the key role in determining the presence of antipox activity.

The most active compounds **13a**–**15a**, **18a**,**b** were investigated for activity against cowpox (CPXV) and ectromelia (ECTV) viruses (Table 2).

For the activity of compounds **13a**–**15a**, **18a**,**b**, the same patterns were obtained as in the case of vaccinia virus, while lower values of the selectivity index were obtained as a result of higher IC_50_ in relation to cowpox and ectromelia viruses. At the same time, as well as the reference compound cidofovir, the compounds showed higher activity against the ectromelia virus than against the cowpox virus.

### 3.4. Molecular Docking

**Figure 6:**
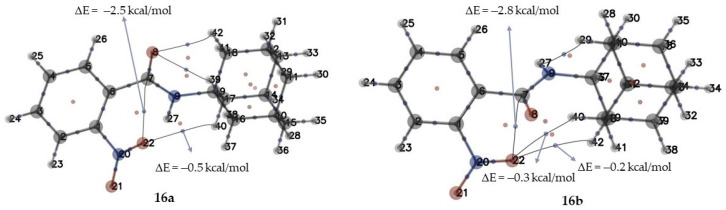
AIM analysis and estimation of hydrogen bonds strength results: two types of critical points are shown: bond critical points (3; −1) correspond to blue points between nuclei; critical points in the ring (3; +1) correspond to red points; ΔE—value of hydrogen bonds strength.

The structures of inactive derivatives **16a**,**b** differ from active derivatives **17**, **18a**,**b** in the nitro group position. Obviously, the activity of investigated compounds relates to their structural features. The presence of a nitro group in the ortho-position could result in the formation of various intermolecular hydrogen bonds between the oxygen atoms -NO_2_ and hydrogen of the adamantane fragment. The results of AIM (atoms in molecules) analysis **16a**,**b** allowed a determination of the bond critical points between the atoms of interest. Very weak dispersion interactions (less than −2.5 kcal/mol) were obtained between pairs of atoms 22 and 40 (Figure 6, compound **16a**), 22–40 and 22–42 (Figure 6, compound **16b**). Weak electrostatic interactions (from −2.5 to −14.0 kcal/mol) were shown between atoms 22 and 7 in both structures. The shown weak dispersion interactions in *ortho*-substituted structures could shield the adamantane fragment or limit the flexibility of molecules **16a**,**b**.

Molecular docking methods were used to evaluate the affinity of the studied structures to the p37 binding site. The choice of the target was based on the structural similarity of investigated compounds **12a**,**b**–**18a**,**b** with derivatives that exhibited antiviral activity against orthopoxviruses due to interaction with p37. In particular, a number of potent antipox agents contained a rigid hydrophobic scaffold (Figure 2 and Scheme 1) [19,20]. We considered the cavity containing Phe52, Leu118, Cys120, Ser135, Asn312, Lys314, Asn329, and Asp331 amino acids as a binding site as it was described in [19,20]. For the molecular docking procedure we used a ligand–protein reference model (Tecovirimat [26,46]—p37), obtained as a result of molecular modeling described in [20].

**Figure 7:**
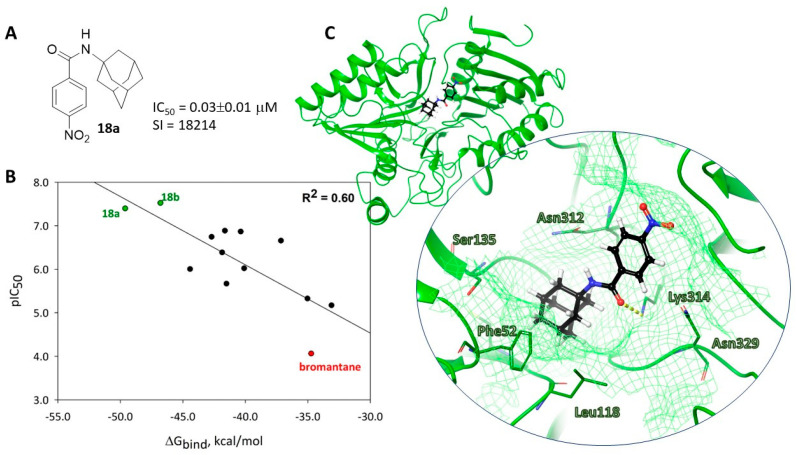
Molecular docking results: (**A**)—lead-compound; (**B**)—correlation between experimental and molecular docking data; (**C**)—location lead-compound in binding site.

All the obtained compounds demonstrated activity against orthopoxviruses except **16a**, which binds the considering cavity with the formation of a series of intermolecular interactions. For active compounds with **18a** being the leader (Figure 7A), realization of the maximum possible number of docking positions was observed (at least 13, Table S3). For inactive **16b**, only five docking positions were registered.

For most compounds, no unfavorable clash interactions were obtained for optimal docking positions except **13a**, **16b**, **17b**, and bromantane. The energy parameters (docking score, Emodel, IFD-score) characterizing the affinities of ligands to the binding site were generally comparable, however, inactive compound **16b** was characterized by the maximum binding energy **Δ**G_bind_. The results of the biological experiments and the binding energies of ligands and protein correlated with an index of 0.60 (Figure 7B). Since the results of the in vitro experiments are the evaluation of antiviral activity only against vaccinia viruses, and not a study of compound affinity to the specific biological target, the obtained value of the correlation index can be considered quite satisfactory.

The most active compound **18a** is located in the binding site with the formation of a hydrogen bridge between the carbonyl oxygen atom of the ligand and the hydrogen atom of the Lys314 NH group. The adamantane fragment of the molecule was surrounded by hydrophobic amino acids such as Phe52, Leu118, Cys12, and Ala134 (Figure 7C).

## 4. Conclusions

A number of adamantane derivatives were obtained; in particular, benzoic acid amides containing an adamantane fragment substituted at the first and second positions were synthesized. Some of the derivatives were not previously described. The structures of most of the obtained compounds were confirmed using the X-ray diffraction method with obtained crystal structures of **12a**–**18a**, **12b**, **13b**, **16b**–**18b**. Almost all compounds had different crystal packings and systems of hydrogen bonds; amides **12a**–**15a** were shown to be isostructural. A number of compounds demonstrated the antiviral activity against vaccinia virus with a selectivity index of up to 18,214 for **18a**. The most active compounds were tested for activity against cowpox (SI = 3036 for **18a**) and ectromelia (SI = 6071 for **18a**) viruses. Molecular modeling of interactions for obtained adamantane derivatives with the p37 viral protein considered as a purposed molecular target was performed at the correlation index of the binding energy and IC_50_ equal to 0.60.

## Figures and Tables

**Table 1:** TC_50_, IC_50_, and SI values for amides **12a**,**b**–**18a**,**b** and bromantane.

Compound	TC_50_, µM ^a^	IC_50_, µM ^b^	SI ^c^
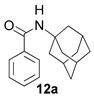	105.67 ± 19.14	4.70 ± 0.37	23
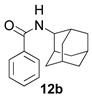	101.33 ± 6.11	6.73 ± 0.17	15
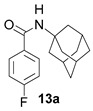	347.31 ± 38.98	0.41 ± 0.10	847
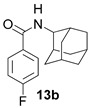	104.00 ± 8.72	2.14 ± 0.13	49
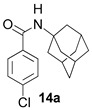	346.00 ± 66.00	0.13 ± 0.08	2662
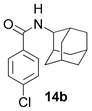	104.00 ± 8.72	0.18 ± 0.04	578
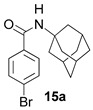	415.00 ± 95.00	0.13 ± 0.08	3192
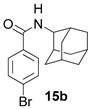	129.67 ± 52.25	0.22 ± 0.07	589
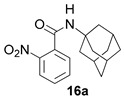	>100	NA ^d^	-
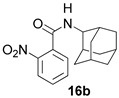	>100	NA ^d^	-
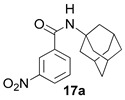	7.75 ± 0.75	0.95 ± 0.03	8
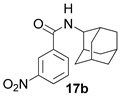	9.50 ± 0.87	0.99 ± 0.19	10
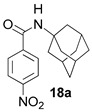	546.43 ± 23.98	0.03 ± 0.01	18,214
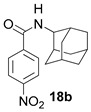	492.00 ± 31.00	0.04 ± 0.01	12,300
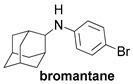	86.60 ± 10.01	38.88 ± 0.49	2
cidofovir	201.33 ± 60.10	7.63 ± 3.15	26

^a^ TC_50_—50% cytotoxic concentration, at which 50% of cells in an uninfected monolayer are destroyed; ^b^ IC_50_—50% virus-inhibiting concentration, at which 50% of cells in the infected monolayer are not destroyed (retain viability); ^c^ SI—selectivity index, SI = TC_50_/IC_50_; ^d^ NA—not active; TC_50_ and IC_50_ values are presented as M ± SD, where M is the mean value, SD is the standard deviation with the number of measurements *n* = 3.

**Table 2:** Antipox activity of **13a**–**15a**, **18a**,**b** against CPXV and ECTV.

Compound	TC_50_, µM ^a^	CPXV		ECTV	
		IC_50_, µM ^b^	SI ^c^	IC_50_, µM	SI
**13a**	347.31 ± 38.98	2.78 ± 0.42	125	1.39 ± 0.29	250
**14a**	346.00 ± 66.00	0.53 ± 0.37	653	0.37 ± 0.33	935
**15a**	415.00 ± 95.00	0.40 ± 0.24	1038	0.15 ± 0.08	2767
**18a**	546.43 ± 23.98	0.18 ± 0.02	3036	0.09 ± 0.017	6071
**18b**	492.00 ± 31.00	1.10 ± 0.22	447	0.45 ± 0.22	1093
cidofovir	201.33 ± 60.10	23.53 ± 2.23	9	6.46 ± 3.10	31

^a^ TC_50_—50% cytotoxic concentration, at which 50% of cells in an uninfected monolayer are destroyed; ^b^ IC_50_—50% virus-inhibiting concentration, at which 50% of cells in the infected monolayer are not destroyed (retain viability); ^c^ SI—selectivity index, SI = TC_50_/IC_50_; TC_50_ and IC_50_ values are presented as M ± SD, where M is the mean value, SD is the standard deviation with the number of measurements *n* = 3.

## Data Availability

Not applicable.

## Supplementary Materials

The following supporting information can be downloaded at: https://www.mdpi.com/article/10.3390/v15010029/s1, Table S1: Crystallographic characteristics, details of the experiments and structure refinement for compounds **12a**–**15a**; Table S2: Crystallographic characteristics, details of the experiments and structure refinement for compounds **16a**–**18a**, **12b**; Table S3: Crystallographic characteristics, details of the experiments and structure refinement for compounds **13b**, **16**–**18b**; Table S4: Molecular docking results; Figure S1. Numeration of atoms in the compounds **16a,b**, **17a,b**, **18b**.
