# Supplementary material for: Structure-Based Design, Synthesis, and Biological Evaluation of the Cage–Amide Derived Orthopox Virus Replication Inhibitors
Source: Viruses. 2022 Dec 21;15(1):29. doi: 10.3390/v15010029 (PMC9865139; doi:10.3390/v15010029)
Supplement: Supplementary file 1 [file viruses-15-00029-s001.zip › viruses-2106802-supplementary.pdf]

Supporting information

Structure-Based Design, Synthesis, and Biological Evaluation of the Cage–Amide Derived Orthopox Virus Replication Inhibitors

Evgenii S. Mozhaitsev [1], Evgeniy V. Suslov [1], Daria A. Rastrepaeva [1], Olga I. Yarovaya [1,*], Sophia S. Borisevich [2], Edward M. Khamitov [2], Dmitry S. Kolybalov [3,4], Sergey G. Arkhipov [3,4,*], Nikolai I. Bormotov [5], Larisa N. Shishkina [5], Olga A. Serova [5], Roman V. Brunilin [6], Andrey A. Vernigora [6], Maxim B. Nawrozkij [7], Alexander P. Agafonov [5], Rinat A. Maksyutov [5], Konstantin P. Volcho [1], and Nariman F. Salakhutdinov [1]

[1] Department of Medicinal Chemistry, N.N. Vorozhtsov Novosibirsk Institute of Organic Chemistry SB RAS, Lavrentyev av. 9, 630090 Novosibirsk, Russia
[2] Laboratory of Chemical Physics, Laboratory of Physical and Chemical methods of analysis, Ufa Institute of Chemistry Ufa Federal Research Center, 71 Pr. Oktyabrya, 450078 Ufa, Russia
[3] Synchrotron Radiation Facility SKIF, G.K. Boreskov Institute of Catalysis SB RAS, 630559 Koltsovo, Russia
[4] Scientific Educational Center "Institute of Chemical Technology", Novosibirsk State University, 630090 Novosibirsk, Russia
[5] State Research Center of Virology and Biotechnology VECTOR, Rospotrebnadzor, 630559 Koltsovo, Russia
[6] Department of Analytical, Physical chemistry and Polymer chemistry and physics, Department of organic chemistry, Volgograd State Technical University Lenina, Avenue 28, 400005 Volgograd, Russia
[7] Center of translational medicine, Sirius University of Science and Technology, Olympic Avenue 1, Krasnodar Krai, 354340 Sirius, Russia; maxim.nawrozkij@gmail.com

List of Content

Figure S1. Numeration of atoms in the compounds 16a,b, 17a,b, 18b 2

Table S1. Crystallographic characteristics, details of the experiments and structure refinement for compounds 12a-15a........ 2

Table S2. Crystallographic characteristics, details of the experiments and structure refinement for compounds 16a-18a, 12b........ 3

Table S3. Crystallographic characteristics, details of the experiments and structure refinement for compounds 13b, 16-18b. 4

Table S4. Molecular docking results........ 5

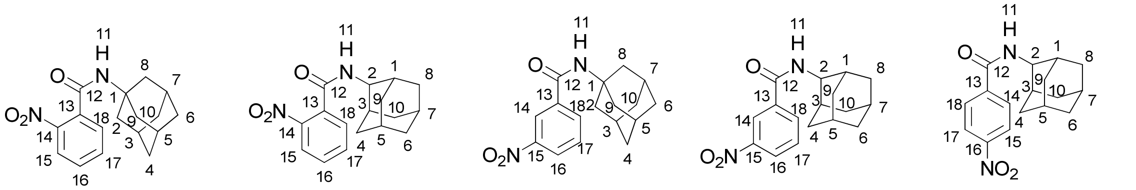

16a **16b** **17a** **17b** **18b**

Figure S1. Numeration of atoms in the compounds **16a,b, 17a,b, 18b.**

Table S1. Crystallographic characteristics, details of the experiments and structure refinement for compounds **12a-15a.**

Parameter	**12a**	**13a**	**14a**	**15a**
Chemical formula	$C_{17}H_{21}NO$	$C_{17}H_{20}FNO$	$C_{17}H_{20}ClNO$	$C_{17}H_{20}BrNO$
M, g/mol	255.35	273.34	289.79	334.25
Temperature, K	99.8(3)	99.95(10)	100.00(10)	100.00(10)
Crystal system	orthorhombic	orthorhombic	orthorhombic	orthorhombic
Space group	*Pbca*	*Pbca*	*Pbca*	*Pbca*
a, Å	11.0252(3)	11.1676(5)	11.4125(5)	11.4460(3)
b, Å	10.3404(3)	10.3102(4)	10.2894(7)	10.2846(4)
c, Å	24.0191(7)	24.0123(11)	24.9460(11)	25.4774(8)
α, deg	90	90	90	90
β, deg	90	90	90	90
γ, deg	90	90	90	90
V, $Å^3$	2738.28(14)	2764.8(2)	2929.4(3)	2999.13(18)
Z	8	8	8	8
ρ_{calc}, g/cm^3	1.239	1.313	1.314	1.481
μ, mm^{-1}	0.076	0.090	0.256	2.737
F(000)	1104.0	1168.0	1232.0	1376.0
Crystal size, mm^3	2.50 × 0.13 × 0.13	0.88 × 0.13 × 0.13	0.75 × 0.50 × 0.13	0.75 × 0.13 × 0.13
2Θ range for data collection, deg	6.786 to 58.846	4.982 to 56.564	3.266 to 65.526	3.198 to 61.046
h, k, l intervals	-15 ≤ h ≤ 14,-14 ≤ k ≤ 13,-32 ≤ l ≤ 32	-14 ≤ h ≤ 14,-13 ≤ k ≤ 13,-31 ≤ l ≤ 31	-17 ≤ h ≤ 15, -15 ≤ k ≤ 14, -27 ≤ l ≤ 37	-16 ≤ h ≤ 13, -14 ≤ k ≤ 14, -36 ≤ l ≤ 36
Measured reflections	43126	44058	17961	32891
Independent reflections [R_{int}, R_σ]	3685 [R_{int} = 0.0504, R_{sigma} = 0.0267]	3425 [R_{int} = 0.0802, R_{sigma} = 0.0418]	5062 [R_{int} = 0.0459, R_{sigma} = 0.0405]	4583 [R_{int} = 0.0471, R_{sigma} = 0.0281]
Data/restraints/parameters	3685/0/235	3425/0/241	5062/0/241	4583/0/241
GOOF on F^2	1.034	1.025	1.051	1.050
R factor ($I>2\sigma(I)$)	R_1 = 0.0442, wR_2 = 0.0951	R_1 = 0.0443, wR_2 = 0.0896	R_1 = 0.0485, wR_2 = 0.1141	R_1 = 0.0343, wR_2 = 0.0744
R factor (all data)	R_1 = 0.0571, wR_2 = 0.1022	R_1 = 0.0763, wR_2 = 0.1037	R_1 = 0.0632, wR_2 = 0.1241	R_1 = 0.0477, wR_2 = 0.0800
$\Delta\rho_{max}$ / $\Delta\rho_{min}$, e/Å^{-3}	0.30/-0.25	0.22/-0.24	0.38/-0.47	0.73/-0.88
CCDC deposition number	2212417	2212421	2213971	2212429

Table S2. Crystallographic characteristics, details of the experiments and structure refinement for compounds **16a-18a, 12b.**

Parameter	**16a**	**17a**	**18a**	**12b**
Chemical formula	$C_{17}H_{20}N_2O_3$	$C_{17}H_{20}N_2O_3$	$C_{17}H_{20}N_2O_3$	$C_{17}H_{21}NO$
M, g/mol	300.35	300.35	300.35	255.35
Temperature, K	100.00(10)	100.00(10)	100.00(10)	100.00(10)
Crystal system	monoclinic	monoclinic	monoclinic	triclinic
Space group	$P2_1/c$	*Pc*	$P2_1/c$	*P*-1
a, Å	7.7346(3)	12.4389(4)	13.1512(7)	10.1202(5)
b, Å	20.4760(5)	11.1070(3)	11.1860(5)	13.9339(6)
c, Å	9.6796(3)	10.7456(3)	10.4483(5)	19.8295(10)
α, deg	90	90	90	89.569(4)
β, deg	108.254(4)	99.211(3)	109.031(6)	89.666(4)
γ, deg	90	90	90	82.020(4)
V, $Å^3$	1455.85(8)	1465.46(7)	1453.03(13)	2769.0(2)
Z	4	4	4	8
ρ_{calc}, g/cm^3	1.370	1.361	1.373	1.225
μ, mm^{-1}	0.095	0.094	0.095	0.075
F(000)	640.0	640.0	640.0	1104.0
Crystal size, mm^3	0.75 × 0.50 × 0.50	0.75 × 0.25 × 0.25	0.75 × 0.13 × 0.13	0.50 × 0.25 × 0.13
2Θ range for data collection, deg	3.978 to 65.62	3.316 to 65.656	3.276 to 58.83	2.952 to 58.564
h, k, l intervals	-11 ≤ h ≤ 11, -30 ≤ k ≤ 30, -14 ≤ l ≤ 14	-18 ≤ h ≤ 18, -9 ≤ k ≤ 16, -16 ≤ l ≤ 15	-18 ≤ h ≤ 17, -15 ≤ k ≤ 15, -14 ≤ l ≤ 13	-10 ≤ h ≤ 13, -17 ≤ k ≤ 18, -25 ≤ l ≤ 25
Measured reflections	30921	17923	24364	25692
Independent reflections [R_{int}, R_σ]	5123 [R_{int} = 0.0323, R_{sigma} = 0.0198]	9411 [R_{int} = 0.0278, R_{sigma} = 0.0481]	3782 [R_{int} = 0.0479, R_{sigma} = 0.0339]	12941 [R_{int} = 0.0345, R_{sigma} = 0.0611]
Data/restraints/parameters	5123/0/259	9411/56/397	3782/0/259	12941/0/685
GOOF on F^2	1.053	1.035	1.046	1.035
R factor ($I>2\sigma(I)$)	R_1 = 0.0387, wR_2 = 0.1022	R_1 = 0.0482, wR_2 = 0.1085	R_1 = 0.0416, wR_2 = 0.0940	R_1 = 0.0565, wR_2 = 0.1128
R factor (all data)	R_1 = 0.0446, wR_2 = 0.1069	R_1 = 0.0575, wR_2 = 0.1157	R_1 = 0.0592, wR_2 = 0.1051	R_1 = 0.0876, wR_2 = 0.1292
$\Delta\rho_{max}$ / $\Delta\rho_{min}$, e/Å^{-3}	0.50/-0.21	0.32/-0.24	0.37/-0.30	0.28/-0.23
CCDC deposition number	2212433	2213998	2212425	2213980

Table S3. Crystallographic characteristics, details of the experiments and structure refinement for compounds **13b, 16-18b.**

Parameter	**13b**	**16b**	**17b**	**18b**
Chemical formula	$C_{17}H_{20}FNO$	$C_{17}H_{20}N_2O_3$	$C_{17}H_{20}N_2O_3$	$C_{17}H_{20}N_2O_3$
M, g/mol	273.34	300.35	300.35	300.35
Temperature, K	100.00(10)	100.00(10)	100.00(10)	100.00(10)
Crystal system	monoclinic	monoclinic	monoclinic	monoclinic
Space group	$P2_1/c$	$P2_1/c$	$P2_1/c$	$P2_1/c$
a, Å	8.6311(6)	7.7552(4)	13.8581(7)	14.0872(9)
b, Å	19.7727(11)	20.7264(9)	11.1634(5)	11.3621(6)
c, Å	8.9580(6)	9.6207(5)	9.8965(4)	9.8245(6)
α, deg	90	90	90	90
β, deg	109.996(7)	111.239(6)	106.485(5)	105.383(7)
γ, deg	90	90	90	90
V, $Å^3$	1436.61(17)	1441.37(14)	1468.10(12)	1516.17(16)
Z	4	4	4	4
ρ_{calc}, g/cm^3	1.264	1.384	1.359	1.316
μ, mm^{-1}	0.087	0.096	0.094	0.091
F(000)	584.0	640.0	640.0	640.0
Crystal size, mm^3	0.50 × 0.25 × 0.25	0.85 × 0.20 × 0.06	0.85 × 0.14 × 0.14	0.50 × 0.25 × 0.13
2Θ range for data collection, deg	4.12 to 65.526	3.93 to 58.918	3.064 to 58.834	4.674 to 65.7
h, k, l intervals	-12 ≤ h ≤ 12, -29 ≤ k ≤ 23, -13 ≤ l ≤ 12	-10 ≤ h ≤ 9, -28 ≤ k ≤ 28, -13 ≤ l ≤ 13	-17 ≤ h ≤ 18, -15 ≤ k ≤ 15, -13 ≤ l ≤ 12	-18 ≤ h ≤ 20, -16 ≤ k ≤ 17, -14 ≤ l ≤ 12
Measured reflections	17492	24415	24834	17930
Independent reflections [R_{int}, R_σ]	4904 [R_{int} = 0.0365, R_{sigma} = 0.0367]	3719 [R_{int} = 0.0465, R_{sigma} = 0.0301]	3785 [R_{int} = 0.0468, R_{sigma} = 0.0355]	5215 [R_{int} = 0.0355, R_{sigma} = 0.0378]
Data/restraints/parameters	4904/0/241	3719/0/259	3785/9/278	5215/0/259
GOOF on F^2	1.028	1.027	1.029	1.027
R factor (I>2$\sigma(I)$)	R_1 = 0.0480, wR_2 = 0.1201	R_1 = 0.0410, wR_2 = 0.0962	R_1 = 0.0453, wR_2 = 0.1024	R_1 = 0.0461, wR_2 = 0.1121
R factor (all data)	R_1 = 0.0655, wR_2 = 0.1345	R_1 = 0.0556, wR_2 = 0.1063	R_1 = 0.0678, wR_2 = 0.1160	R_1 = 0.0650, wR_2 = 0.1257
$\Delta\rho_{max}$ / $\Delta\rho_{min}$, e/Å^{-3}	0.42/-0.27	0.33/-0.25	0.33/-0.29	0.36/-0.22
CCDC deposition number	2212423	2212435	2212430	2212428

Table S4. Molecular docking results.

Ligand	pIC50	Pose (max 20)	Docking score	Emodel	IFD	H-bond	Other interactions	Clash	ΔG$_{bind}$
**12a**	5.33	16	-6.456	-52.551	-586.52	K314	L118; C120; A134; F52 - HB	none	-35.03
**12b**	5.17	16	-6.464	-55.619	-583.54	K314; N329	F52; L118; C120; A134 - HB	none	-33.10
**13a**	6.39	19	-7.12	-55.503	-590.83	S140, N312		S135	-41.85
**13b**	5.67	17	-7.504	-60.705	-585.19	S140; N312	A134; A328; L118; C120; F52; C53 – HB	none	-41.51
**14a**	6.87	13	-6.766	-57.953	-586.8	K314	A134; F52; L118; C120 - HB	none	-40.37
**14b**	6.74	18	-7.50	-60.927	-585.75	S140; N312		none	-42.70
**15a**	6.89	18	-6.76	-60.756	-588.59	K314	L118; A328; C120; F55 - HB	none	-41.63
**15b**	6.66	17	-6.456	-59.044	-585.55	K314	A134; F52; L118; C120 - HB	none	-37.15
**16a**	n/a	No docking results							
**16b**	n/a	5	-6.448	-56.451	-582.32	K134; N329	F52 C53; L118; A134; - HB	N321	-20.50
**17a**	6.02	18	-6.92	-63.58	-589.4	K314; K281	K281 - salt bridge; A134; F52; C120; L118 – HB	none	-40.09
**17b**	6.00	18	-7.8	-63.126	-589.06	S140, N312		none	-44.42
**18a**	7.52	18	-7.02	-63.66	-588.78	K314	L118; A328; C120; F52 - HB	none	-46.80
**18b**	7.40	18	-7.596	-61.88	-587.65	N312; S140	A134; C120; L118; A328 - HB	S327	-49.62
**bromantane**	4.06	18	-6.666	-50.79	-586.72		F52; C53; A328 – HB	T137	-34.73
